# Mechanistic Insights into Multiherb Formulations for Antibiotic-Associated Diarrhea: A Systematic Review of Preclinical Studies on Microbiome–Host Interactions

**DOI:** 10.3390/ijms27083663

**Published:** 2026-04-20

**Authors:** Ji Hye Hwang, You-Kyung Choi

**Affiliations:** 1 Department of Acupuncture & Moxibustion Medicine, College of Korean Medicine, Gachon University, Seongnam 13120, Republic of Korea; jhbori@nate.com; 2 Department of Korean Internal Medicine, College of Korean Medicine, Wonkwang University, Iksan 54538, Republic of Korea

**Keywords:** antibiotic-associated diarrhea, gut microbiome, multiherb formulations, intestinal barrier

## Abstract

Antibiotic-associated diarrhea (AAD) is primarily driven by disruption of the gut microbiota accompanied by intestinal mucosal injury. Although multiherb formulations are widely used in East Asian medicine, their collective ecological effects and integrated microbiome–host mechanisms have not been systematically synthesized. This systematic review included 17 preclinical studies that investigated multiherbal formulations in AAD models. Given the substantial heterogeneity in the formulation composition, experimental design, and analytical platforms, a descriptive synthesis was performed. The included formulations were categorized into four clusters based on their shared herbal composition: Qiwei Baizhu San (QWBZP), Lizhong Tang (LZT), Gegen Qinlian Tang (GQT), and other supportive multiherbal formulations. The cluster-based synthesis revealed distinct convergent therapeutic strategies. The QWBZP and LZT clusters primarily supported the restoration of host metabolic and digestive functions, whereas the GQT cluster exhibited potent pathogen control effects with the suppression of opportunistic taxa. Across all clusters, a convergent microbiome–host response emerged, characterized by enrichment of commensal bacteria (e.g., *Lactobacillus*), upregulation of tight junction proteins (e.g., ZO-1, occludin), and attenuation of pro-inflammatory mediators (e.g., TNF-α, myeloperoxidase). Multiherb formulations in AAD models not only act as microbial modulators but also function as host-directed modulators that stabilize the intestinal homeostatic niche. Botanical interventions may facilitate endogenous microbiome recovery by reinforcing mucosal integrity and reducing environmental resistance. This ecological framework provides a rationale for future translational studies evaluating integrated herbal–probiotic strategies and precise microbiome management for patients with AAD, while further clinical validation is warranted.

## 1. Introduction

Antibiotic-associated diarrhea (AAD) is a common adverse effect of antibiotic therapy resulting from the disruption of the gut microbiota and impairment of intestinal homeostasis [1,2]. In addition to transient gastrointestinal symptoms, AAD can predispose patients to prolonged dysbiosis, impaired mucosal barrier function, and secondary infections, including *Clostridioides difficile* infection [3,4]. Increasing evidence indicates that alterations in microbial diversity, metabolic capacity, and host–microbiota interactions are considered to play central roles in the pathogenesis and persistence of AAD [5,6].

Various therapeutic strategies have been explored to mitigate AAD, including probiotics, prebiotics, and dietary interventions [1,7,8]. Probiotic-based approaches aim to restore microbial balance through supplementation with selected strains; however, their efficacy remains variable and strain-specific, and direct evidence supporting consistent clinical benefit remains limited, with ongoing concerns regarding safety and colonization resistance in vulnerable populations [7,9]. Herbal medicines have been commonly used in East Asian clinical practice for the treatment of gastrointestinal disorders, including antibiotic-related diarrhea, often in the form of multiherb formulations rather than as single botanical components [10,11].

Multiherbal formulations represent a distinct therapeutic paradigm characterized by combinatorial botanical compositions and multi-target actions, reflecting long-standing theories of herb–herb combinations in traditional East Asian Medicine [10,11,12]. Unlike single-compound interventions, these formulations are thought to modulate microbial composition, metabolic function, and host responses [11]. Experimental studies have suggested that such formulations may influence gut microbiota structure and short-chain fatty acid production [13,14,15,16,17,18,19,20,21,22], as well as intestinal barrier integrity and inflammatory signaling [16,23,24,25,26,27,28,29]. Recent studies have further emphasized the importance of microbiome-targeted interventions in antibiotic-associated diarrhea, highlighting both probiotic-based strategies and emerging integrative approaches that simultaneously modulate microbial composition and host responses [30]. Nevertheless, the inherent complexity of multiherb prescriptions poses substantial challenges for evidence synthesis, particularly when studies differ in formulation composition, processing methods, and analytical platforms.

Previous systematic reviews in this field, including our recent evaluation of single botanical agents [31], have predominantly focused on single components or probiotic-based interventions, with limited attention paid to multiherbal formulations as a distinct category. Consequently, it remains unclear whether convergent patterns exist across heterogeneous preclinical studies of multiherb prescriptions and how such patterns relate to microbiome- and host-related outcomes in AAD models.

To address this gap, the present systematic review synthesized preclinical animal studies investigating multiherbal formulations for AAD. Rather than attempting a quantitative meta-analysis, we applied a cluster-based descriptive approach by grouping the formulations according to their shared herbal compositions. This strategy aimed to clarify recurrent outcome patterns across formulation clusters, integrate microbiome- and host-related findings, and critically assess the methodological heterogeneity and risk of bias. In doing so, this review provides a structured framework for interpreting complex preclinical evidence and will inform future experimental designs for the study of multiherbal interventions for AAD. This approach also enables interpretation of microbiome–host interactions within a mechanistic and ecological context.

## 2. Methods

### 2.1. Search Strategy

**Figure 1:**
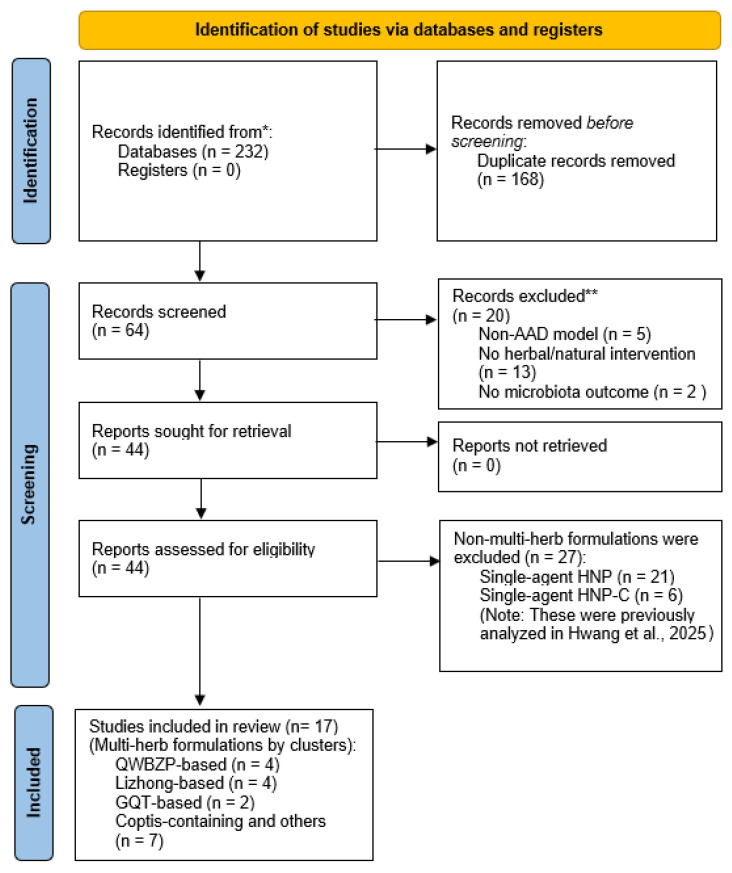
PRISMA 2020 flow diagram of the study selection process. Of the 44 reports assessed for eligibility, 27 studies focusing on single-agent interventions (HNP and HNP-C) are excluded to avoid redundancy, as they are independently analyzed in our previous systematic review [31]. Consequently, 17 studies investigating multiherb formulations are included and categorized into four strategy-based clusters for qualitative synthesis. HNP, herbal and natural products (single-agent interventions); HNP-C = herbal and natural products co-administered with probiotics or synbiotics; QWBZP, Qiwei Baizhu San; and GQT, Gegen Qinlian Tang. * Databases searched include PubMed, EMBASE, Web of Science, Scopus, and CNKI, from their inception. ** Records were excluded based on title and abstract screening for the following reasons: non-AAD model (n = 5); no herbal/natural intervention (n = 13); no microbiota outcome (n = 2).

The methodology for this review was prespecified and informed by a previously published protocol [32], which outlined the study design and inclusion criteria. This study was conducted in accordance with the Preferred Reporting Items for Systematic Reviews and Meta-Analyses (PRISMA) guidelines [33], and the study selection process is illustrated in Figure 1. Data were obtained from a comprehensive systematic search of major international and regional databases, including PubMed, EMBASE, Web of Science, Scopus, and CNKI, from their inception. This search was conducted as part of a larger project investigating herbal interventions for AAD (PROSPERO CRD420251136553). While the single-herb component of this project has been reported previously [31], the present review specifically focuses on multiherbal formulations and analyzes studies reporting integrated microbiome- and host-related outcomes, thereby addressing a distinct research objective.

### 2.2. Eligibility Criteria

Studies were included in this review if they satisfied the following criteria:

Population: In vivo animal models of AAD with no restrictions on species, sex, age, or body weight.
Intervention: Multiherb formulations (MHF) consisting of two or more distinct medicinal herbs, regardless of the extraction method or dosage.
Comparison: Control groups receiving drug vehicle, untreated AAD model controls, antibiotic monotherapy, or established positive controls (e.g., probiotics used as an independent comparator arm).
Outcomes: Quantitative assessment of gut microbiota composition via 16S rRNA sequencing, metagenomic profiling, or targeted molecular assays (e.g., qPCR), reporting diversity indices α and β, and taxonomic shifts.
Study design: Peer-reviewed controlled in vivo studies with available full-text articles.
Studies were excluded if they focused exclusively on single chemical compounds, herbal interventions, or probiotic-only treatments.

### 2.3. Study Selection and Data Extraction

Two reviewers independently screened the titles and abstracts, and then evaluated the full-text article according to the predefined eligibility criteria. Discrepancies were resolved by discussion or consultation with a third reviewer. Data were extracted independently using a standardized form, including: (1) animal characteristics (species and strain); (2) AAD induction protocol; (3) treatment formulation composition; (4) comparator arms and treatment duration; (5) microbiome analytical platforms; (6) microbiota diversity and taxonomic changes; and (7) host-related outcomes (short-chain fatty acids [SCFAs], intestinal barrier markers, inflammatory cytokines, and digestive enzymes). All data were extracted as reported in the original articles, without imputation.

### 2.4. Assessment of Risk of Bias

The risk of bias in the included studies was assessed using the SYRCLE Risk of Bias (RoB) tool for animal studies [34]. Each study was evaluated across 10 domains: selection bias (sequence generation, baseline characteristics, and allocation concealment), performance bias (random housing and blinding), detection bias (random outcome selection and blinding), attrition bias, reporting bias, and other potential sources of bias. Each domain was rated as having a low, unclear, or high risk of bias based on explicit reporting in the original studies.

### 2.5. Data Synthesis and Analysis

Owing to the substantial methodological heterogeneity across animal models, antibiotic protocols, and herbal compositions, a quantitative meta-analysis was not performed. Instead, qualitative synthesis was conducted using a strategy-based clustering approach. The multiherb formulations were categorized into four distinct clusters based on their core herbal ingredients and traditional therapeutic principles (spleen-tonifying, middle-warming, and heat-clearing). To identify the common botanical drivers within these clusters, the frequency of individual herbal components was calculated for all the included formulations. This integrated approach allowed the identification of convergent microbiome–host response patterns specific to each herbal treatment strategy.

## 3. Results

### 3.1. Study Characteristics and Formulation Composition

Seventeen animal studies investigating multiherbal formulations for AAD were included [13,14,15,16,17,18,19,20,21,22,23,24,25,26,27,28,29] (Table 1). All studies employed rodent models of antibiotic-induced diarrhea using mice or rats with heterogeneous antibiotic regimens and induction periods. The treatment duration ranged from 3 to 7 days, with a total experimental period of 8 to 21 days, depending on the model design.

The included interventions comprised classical multiherbal formulations and their modified or fractionated forms. The herbal compositions were explicitly reported in all studies and are summarized in Table 1. The number of constituent herbs per formulation ranged from two to seven, reflecting diverse formulation principles and therapeutic intentions. Comparator arms commonly included untreated normal controls and AAD model controls, whereas several studies additionally incorporated alternative comparators, such as fermented formulations or single-herb controls.

Microbiome-related outcomes were assessed using heterogeneous analytical platforms. Most studies employed 16S rRNA sequencing, while others used methods such as targeted functional gene analysis, T-RFLP, or shotgun metagenomics. This methodological diversity precluded direct quantitative comparisons across studies but enabled a broad descriptive synthesis of microbiome-related outcomes.

### 3.2. Clustering of Formulations Based on Herbal Composition and Shared Core

Despite the substantial heterogeneity in the experimental design, several formulation clusters were identified (Table 1). Subsequent frequency analysis (Table 2) revealed that *Atractylodes macrocephala* (70.6%), *Glycyrrhiza uralensis* (64.7%), and *Poria cocos* (58.8%) were the three most frequently incorporated components, appearing in over 58% of the studies as a shared pharmacological core. This recurrent co-occurrence suggests the presence of a common formulation logic underlying multiple prescription clusters, rather than isolated botanical effects.

The specific combinations of these herbs reflect distinct therapeutic strategies, beginning with the QWBZP-based cluster (n = 4), which included the original decoction, glycoside fractions, and sucrose-modified forms [13,14,15,16]. Beyond the shared core, this cluster was characterized by the addition of aromatic and fluid-regulating herbs, such as *Aucklandia lappa, Agastache rugosa,* and *Pueraria lobata*. Traditionally aimed at strengthening the spleen and stopping diarrhea, studies within this cluster have predominantly reported functional recovery of digestion, as evidenced by improved lactase activity.

In contrast, the LZT-based cluster (n = 4), encompassing whole decoctions and polysaccharide fractions, was distinguished by the essential inclusion of Zingiberis Rhizoma (Dried Ginger) [17,18,19,20]. This defines its role in warming the middle and dispelling cold, targeting metabolic stabilization and the restoration of microbial diversity from a “cold-type” functional suppression state.

Furthermore, the GQT and Coptis-containing clusters (n = 5), which evaluated both the classical GQT formula and non-identical prescriptions such as the Xianglian Pill and the Shengjiang Xiexin Decoction, utilized *Coptis chinensis* as a shared component [21,22,23,24,25]. These formulations collectively focused on clearing heat and resolving dampness, showing a strong capacity to suppress Proteobacteria and reduce inflammatory mediators like TNF-α.

Finally, the other multiherb formulations (n = 4) included various prescriptions focused on reinforcing the mucosal barrier and reducing acute injury markers like myeloperoxidase (MPO) activity, thereby preventing bacterial translocation through Qi-regulating and barrier-protecting mechanisms [26,27,28,29]. Although overlapping herbal components were observed across these clusters, differences in the formulation composition, extraction methods, dosages, and microbiome analytical platforms limited further quantitative integration. Accordingly, the formulations were synthesized descriptively rather than by a compound–target network analysis. Based on these defined clusters, microbiome- and host-related outcomes were examined at both the individual study and cluster levels.

### 3.3. Study-Level Microbiome and Host Outcomes

Across the 17 included studies [13,14,15,16,17,18,19,20,21,22,23,24,25,26,27,28,29], multiherb formulations demonstrated heterogeneous but partially convergent effects on the composition of the gut microbiota and host-related outcomes within the scope of this review (Table 3). However, these findings should be interpreted with caution, as the analysis was restricted to studies reporting microbiome-related outcomes, which may limit the generalizability of the conclusions. Most studies reported an increase in α-diversity following herbal intervention compared with AAD controls, with the notable exception of one QWBZP-based study [13], in which α-diversity decreased, likely reflecting selective dominance of lactase-positive bacteria rather than global microbial expansion.

β-diversity analyses, assessed using principal coordinate analysis (PCoA), non-metric multidimensional scaling (NMDS), or related ordination methods, consistently demonstrated a shift away from the dysbiotic AAD state toward normal controls across formulation clusters [15,16,17,18,19,20,21,22,23,24,25,27]. Studies employing non-NGS platforms, such as T-RFLP or functional gene-based PCR assays, have reported OTU recovery or functional realignment rather than taxonomic clustering [13,14,26], underscoring methodological heterogeneity.

Taxonomic changes varied by formulation but converged on reduced dysbiosis-associated taxa. Increases in *Lactobacillus*, *Firmicutes*, and *Bacteroides*-related genera and decreases in Proteobacteria and *Clostridioides difficile* were commonly observed across studies, although genus-level resolution was not uniformly available.

Direct quantification of SCFAs was reported in only two studies using GC–MS [16,23], both of which demonstrated increased SCFA levels following treatment. Other studies indirectly inferred SCFA-related effects through the enrichment of SCFA-associated taxa or functional gene markers [17,24].

Barrier-related outcomes as well as immune and inflammatory markers were reported. Improvements in mucosal integrity markers (e.g., ZO-1, occludin, and MUC2) were observed in several studies [16,23,25,26,27], reductions in pro-inflammatory cytokines (e.g., TNF-α and IL-17) were reported in others [16,23,25], and inhibition of bacterial translocation was described in selected formulations [26,28]. Digestive enzyme activity, including lactase and sucrase activities, was assessed in only two formulations [13,29], highlighting the recovery of digestive function as a formulation-specific rather than a universal outcome.

### 3.4. Integrated Outcome Patterns Across Formulation Clusters

When the outcomes were summarized at the formulation cluster level (Table 4), multiherb formulations exhibited cluster-dependent patterns across the microbiome- and host-related domains.

QWBZP-based formulations [13,14,15,16] demonstrated consistent alterations in microbial functions related to carbohydrate digestion. Although the global α-diversity did not uniformly increase within this cluster [13], enrichment of lactase-positive bacteria and increased lactase activity or gene abundance were repeatedly reported [13,14,16], indicating selective functional modulation rather than broad expansion of microbial diversity.

LZT-based formulations [17,18,19,20] primarily showed restoration of microbiota composition, as reflected by recovery of β-diversity profiles, increases in *Firmicutes*-associated taxa, and reductions in dysbiosis-related taxa such as Proteobacteria or *Clostridioides difficile* [17,18,19,20]. However, direct measurements of SCFAs and barrier-related outcomes were limited to this cluster [20].

GQT-based studies [21,22] consistently reported shifts in β-diversity and enrichment of commensal bacterial taxa. A study employing shotgun metagenomics [22] further identified alterations in functional pathways and reductions in antibiotic resistance gene abundance, suggesting functional changes beyond taxonomic restructuring.

Formulations containing *Coptis chinensis* [23,24,25] but not sharing identical prescriptions demonstrated relatively consistent host-related outcomes. These included direct increases in SCFA levels [23], enhanced expression of tight junction-related markers [24,25], and reductions in pro-inflammatory cytokines [23,25], although such outcomes were not uniformly assessed across all studies.

Other multiherb formulations [26,27,28,29] showed heterogeneous but directionally similar patterns, including increased α-diversity, shifts toward microbial communities resembling normal controls, and improvements in selected host-related indicators such as bacterial translocation or inflammatory markers [26,28].

Given the heterogeneity in the formulation composition, analytical platforms, and outcome reporting, these findings were synthesized descriptively. No quantitative meta-analyses or compound–target network analyses were performed. In addition to standard comparisons with untreated AAD model controls, a limited number of studies incorporated modified formulations or processing-based comparators, including fermented preparations [27], fractionated extracts [15,20], and formulations supplemented with fermentable substrates [16] (Table 4).

### 3.5. Integrated Outcome Patterns: The Microbiome–Host Axis

Across formulation clusters, the therapeutic effects of multiherb interventions in AAD models were not confined to microbial compositional changes but were accompanied by coordinated improvements in host-related outcomes. In several studies, enrichment of commensal genera such as *Lactobacillus*, *Bacteroides*, and other *Firmicutes* was observed in parallel with markers of intestinal barrier stabilization and attenuation of inflammatory responses [16,20,23,24,25,26,28].

Notably, studies reporting microbial enrichment also documented enhanced barrier integrity, reflected by increased expression of tight junction-related markers, including ZO-1, occludin, and MUC2 [16,23,25,27], as well as reduced bacterial translocation [26,28]. In addition, a subset of studies demonstrated suppression of pro-inflammatory mediators, such as TNF-α, IL-17, and MPO, suggesting a coordinated host response following microbial modulation [16,23,25,28].

Although direct causal relationships could not be established from the included studies, these convergent findings suggest the presence of an integrated microbiome–host axis in multiherb-treated AAD models. Collectively, these results indicate that multiherbal formulations may exert therapeutic effects through the simultaneous restructuring of the gut microbiota and functional stabilization of the intestinal environment, rather than through isolated antimicrobial actions.

### 3.6. Risk of Bias Results

**Figure 2:**
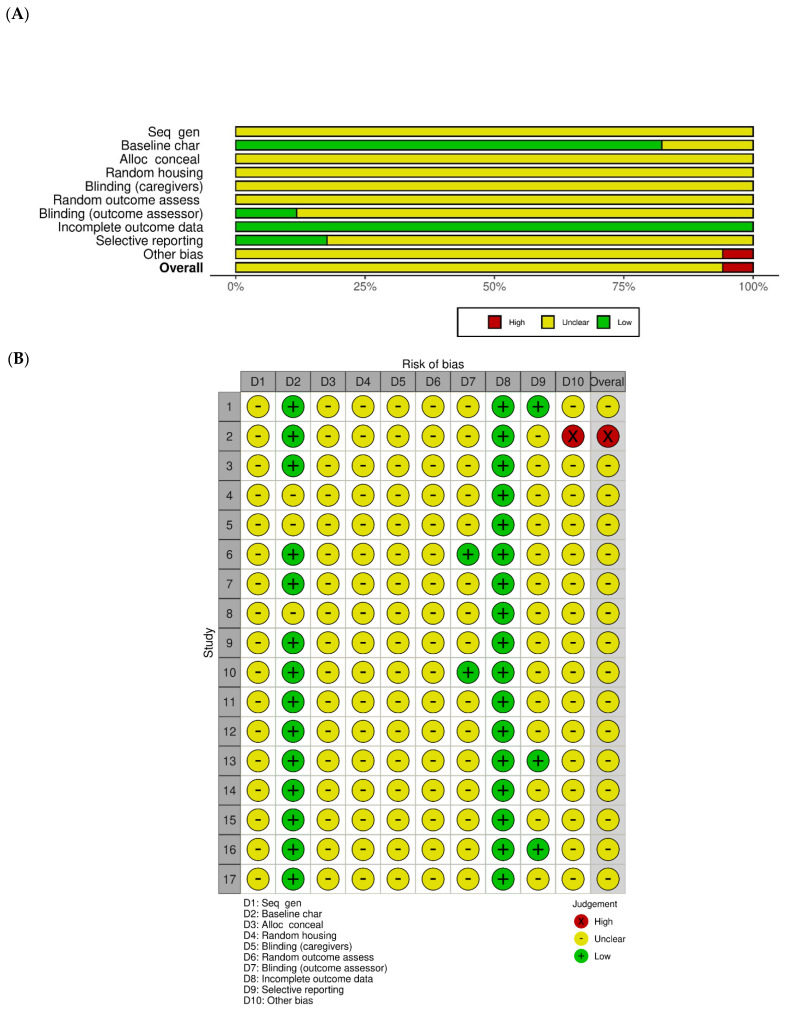
SYRCLE risk-of-bias assessment of included animal studies: (**A**) Summary of risk-of-bias ratings across all SYRCLE domains, presented as the percentage of studies classified as low, high, or unclear risk. (**B**) Risk-of-bias evaluation for each individual study (n = 17), with SYRCLE items scored as low risk (yes), high risk (no), or unclear risk (unclear). SYRCLE: Systematic Review Centre for Laboratory Animal Experimentation. D1–D10: individual SYRCLE risk-of-bias domains, including sequence generation, baseline characteristics, allocation concealment, random housing, blinding (caregivers and outcome assessors), incomplete outcome data, selective reporting, and other sources of bias.

Risk of bias was assessed using the SYRCLE risk of bias tool for animal studies [13,14,15,16,17,18,19,20,21,22,23,24,25,26,27,28,29] (Figure 2). Most studies adequately reported baseline characteristics, including animal strain, age, body weight, and group allocation and were therefore judged to have low risk of bias in this domain. Incomplete outcome data were generally judged as low risk across studies, as planned euthanasia schedules were reported and no substantial attrition was observed.

However, sequence generation and allocation concealment were frequently insufficiently described, resulting in unclear risk of bias for these domains in most studies. Similarly, random housing and the blinding of caregivers or outcome assessors were rarely reported. Selective outcome reporting was judged as low-risk in a subset of studies in which all prespecified outcomes were consistently reported, whereas the remaining studies were rated as unclear due to the absence of protocol information.

Overall, most of the included studies were assessed as having unclear to moderate risk of bias, primarily due to incomplete reporting rather than explicit methodological flaws.

## 4. Discussion

### 4.1. Principal Findings

This systematic review synthesized preclinical evidence from 17 animal studies investigating multiherb formulations for the treatment of AAD. Unlike previous reviews that focused on single components or probiotic-based interventions, the present study specifically addressed the complexity of multiherbal prescriptions by organizing heterogeneous formulations into clusters based on shared herbal compositions.

Across clusters, the multiherbal formulations generally ameliorated antibiotic-induced dysbiosis and were associated with improvements in microbiome- and host-related outcomes. Although the experimental designs and analytical platforms varied substantially, convergent patterns were observed, suggesting that multiherb formulations exert therapeutic effects through coordinated modulation of the gut ecosystem rather than through isolated antimicrobial activity (Table 3).

### 4.2. Interpretation of Cluster-Based Findings

Clustering formulations according to the herbal composition enabled the structured synthesis of otherwise heterogeneous studies, revealing how specific botanical combinations drive distinct therapeutic outcomes. The QWBZP-based formulations [13,14,15,16] primarily exhibited selective functional modulation of the microbiota, particularly in pathways related to carbohydrate digestion. Rather than uniformly increasing the global microbial diversity, these formulations frequently enriched lactase-positive bacteria and enhanced lactase activity [13,14,16]. This functional recovery was likely driven by potential synergistic interactions between *Panax ginseng* and *Atractylodes macrocephala*. Within this cluster, the qi-tonifying properties of ginseng provided the metabolic signals and energy required for rapid mucosal restitution by promoting intestinal epithelial cell migration [35]. The combined use of Atractylodes and Ginseng has been shown to exert superior effects on gut microbial modulation and mucosal protection compared to single agents [36]. Furthermore, *Poria cocos* optimizes nutrient transport and transformation by regulating brain–gut peptides and repairing the gastrointestinal mucosa [37], collectively restoring luminal digestive capacity.

LZT-based formulations [17,18,19,20] predominantly demonstrated recovery of the microbial community structure, as reflected by the normalization of β-diversity profiles and reductions in dysbiosis-associated taxa such as *Proteobacteria* [17,18,19,20]. The core strategy of “warming the middle” in this cluster was facilitated by the essential combination of Zingiberis Rhizoma (Dried Ginger) and *Panax ginseng*. *Ginseng* acts as a systemic bioactivator that supports the thermal and metabolic effects of ginger, collectively helping to counteract antibiotic-induced functional suppression and restore the intestinal barrier and microbial balance [38]. This creates a physiological environment conducive to the reestablishment of commensal *Firmicutes*, although direct evidence of SCFAs remains limited in this cluster, except for studies focusing on polysaccharide fractions [20].

Collectively, the findings from GQT-based studies [21,22] in AAD models indicated consistent β-diversity shifts and suppression of dysbiosis-associated taxa. These findings are conceptually aligned with the traditional “heat-clearing” and “detoxifying” functions of *Coptis chinensis* and *Scutellaria baicalensis*, which have been reported to modulate gut microbial composition and inflammatory responses through microbiota–metabolite interactions [39]. Mechanistic evidence from experimental studies in inflammatory and metabolic disease models further supports this interpretation, demonstrating that GQT and related Scutellaria–Coptis formulations can modulate gut microbial composition, enhance SCFA production, stabilize intestinal barrier function, and attenuate excessive inflammatory signaling [40,41,42]. Notably, one of the included AAD studies employing shotgun metagenomics identified functional pathway alterations following GQT treatment [22], suggesting that its therapeutic effects may extend beyond taxonomic restructuring toward functional reprogramming of the gut ecosystem. Although direct evidence for antibiotic resistance gene modulation remains limited within AAD models, these convergent findings provide a biologically plausible framework that supports the role of GQT in restoring intestinal homeostasis under antibiotic-induced stress.

Taken together, the GQT-based studies [21,22] and Xianglian Pill [23] can be collectively interpreted as representing a “pathogen control-oriented” therapeutic strategy, characterized by suppression of opportunistic and inflammation-associated taxa following antibiotic exposure. This shared pattern is plausibly attributable to the high berberine content of Coptis chinensis, which has been widely reported to exert antimicrobial and microbiota-modulating effects under dysbiotic conditions [43].

In contrast, the Shengjiang Xiexin Decoction (SJT) [24,25] represents a more integrative therapeutic strategy that combines pathogen suppression with harmonization of the intestinal environment. Although the inclusion of *Coptis chinensis* and *Scutellaria baicalensis* contributes to antimicrobial and anti-inflammatory effects, the SJT simultaneously incorporates warming and harmonizing components such as Zingiberis Rhizoma, Pinelliae Rhizoma, and *Panax ginseng* [43,44]. Rather than acting as simple adjuncts, these components have been traditionally and experimentally associated with the stabilization of gastrointestinal function under stress or inflammation [45,46]. Experimental studies examining *Clostridioides difficile* infection and chemotherapy-induced diarrhea models have demonstrated that SJT can modulate gut microbiota composition, bile acid metabolism, and mucosal inflammatory responses, thereby alleviating infection- or treatment-associated intestinal dysfunction [45,46,47,48]. Taken together, this composite formulation illustrates a dual-action profile in which the suppression of inflammatory dysbiosis is accompanied by the mitigation of functional disturbance, potentially enabling a more comprehensive stabilization of the host–microbiome axis than formulations focused predominantly on pathogen control.

Despite their compositional heterogeneity, the remaining multiherbal formulations [26,27,28,29] demonstrated directionally consistent host-related outcomes. These studies commonly reported the reinforcement of intestinal barrier integrity and attenuation of injury-related markers, such as reduced bacterial translocation, decreased MPO activity, and upregulated expression of tight junction proteins. Rather than representing a unified prescription cluster, these formulations functionally converge on a barrier reinforcement strategy that facilitates mucosal stabilization under antibiotic-induced stress. Notably, although these formulations lacked an identical prescription structure, *Pericarpium citri reticulatae* was identified as a common constituent in 75% (3 of 4) of the studies within this group [26,28,29]. This observation does not imply a direct causal role of *Citri Unshius Pericarpium* on its own but rather highlights a recurrent botanical feature that may contribute to the observed mucosal recovery. The inclusion of *Citri Unshius Pericarpium* in these formulations aligns with its traditional role of “regulating Qi,” a concept historically associated with the alleviation of gastrointestinal stagnation and functional disharmony. Although this classical concept has not been mechanistically defined, emerging experimental evidence has provided a plausible biological parallel. For instance, naringenin, a key flavonoid derived from *Citri Unshius Pericarpium*, has been shown to mitigate barrier disruption by restoring occludin and claudin-1 expression via inhibition of NF-κB–mediated pathways [49]. Consistently, our results suggest that formulations containing Qi-regulating components are frequently associated with physical barrier reinforcement, including the upregulation of MUC2 observed in this cluster. This integration of classical therapeutic principles with modern molecular evidence warrants further mechanistic investigation of how *Citri unshius pericarpium* may contribute to the stabilization of the microbiome–host axis.

### 4.3. Integrated Microbiome–Host Response Patterns

Beyond cluster-specific findings, an integrated pattern linking microbiome modulation and host recovery emerged across the reviewed studies (Table 3). The enrichment of commensal genera, such as *Lactobacillus*, *Bacteroides*, and members of *Firmicutes*, was frequently accompanied by improvements in intestinal barrier integrity [16,23,25,26,27], reduced bacterial translocation [26,28], and attenuation of mucosal or systemic inflammatory responses [16,23,25,28]. Several studies have reported increased expression of tight junction-related proteins (e.g., ZO-1, occludin, and MUC2) [16,23,25,27], together with reduced bacterial translocation following multiherb interventions [26,28]. In parallel, decreases in pro-inflammatory mediators, including TNF-α, IL-17, and MPO activity, were observed across multiple formulation clusters [16,23,25,28].

Direct causal relationships cannot be established, although these convergent observations suggest a coordinated therapeutic response potentially targeting the integrated microbiome–host axis. Conceptually, this pattern is consistent with a “soil-priming” effect, whereby botanical interventions stabilize the host’s physiological environment, such as mucosal integrity, inflammatory tone, and metabolic capacity, to facilitate the successful engraftment of commensal microbial “seeds” [50]. From an ecological perspective, such host-mediated modulation may reduce colonization resistance and shape microbial invasion dynamics, thereby promoting a more resilient gut ecosystem [51]. Taken together, these findings suggest that multiherb formulations may support long-term ecological restoration in AAD models not by enforcing microbial replacement, but by creating a permissive host environment that enables endogenous microbiome recovery.

### 4.4. From Competition to Synergy: Rationale for Integrated Herbal–Probiotic Strategies

The findings of this review suggest a conceptual shift in the development of therapeutic strategies for AAD, moving beyond a competition-based framework toward a synergy-oriented approach. In most of the included studies, probiotics were incorporated primarily as active comparators to benchmark the efficacy of multiherb formulations. Although this design has been useful for efficacy validation, it inherently reflects an “either–or” perspective that may overlook potential complementary interactions between these two modalities.

A recognized limitation of probiotic monotherapy for AAD is the low and inconsistent colonization efficiency of exogenously administered strains in an antibiotic-disrupted intestinal environment [7,9].

Antibiotic-induced dysbiosis is frequently accompanied by impaired mucosal integrity, altered digestive capacity, and increased inflammatory stress, all of which may constrain microbial survival and function. Notably, the present synthesis indicates that multiherb formulations exert pronounced host-directed effects that extend beyond microbial compositional shifts.

As summarized in Table 3, formulations containing *Coptis chinensis* were associated with increased expression of tight junction-related markers (e.g., ZO-1 and occludin) [23,24], whereas selected multiherb formulations reduced indicators of mucosal injury and inflammation, such as MPO activity [28]. In addition, a subset of the formulations enhanced digestive enzyme activity [13,29], suggesting a partial restoration of luminal functional capacity. Collectively, these findings are consistent with the hypothesis that multiherb interventions contribute to “soil conditioning” by stabilizing the structural and immunological features of the intestinal niche, which is increasingly being emphasized in next-generation microbiome-based strategies [52].

From this perspective, multiherb formulations can be conceptualized as biological primers that improve host readiness, rather than direct microbial replacement. Although probiotic colonization or persistence was not directly assessed in the included studies, the observed improvements in barrier integrity (e.g., ZO-1 and MUC2 expression) [16,23,25] and digestive function [13,14] provide a biological rationale for the hypothesis that such host-level changes could create a more permissive environment for microbial survival and activity. However, direct evidence for enhanced probiotic engraftment remains limited.

### 4.5. Conceptual Framework and Future Directions

Based on these considerations, this review proposes an integrated herbal–probiotic framework, in which multiherbal formulations and probiotics serve complementary rather than redundant roles. Within this framework, probiotics function as exogenous microbial “seeds,” whereas multiherb formulations act as biological primers that stabilize the host “soil” by reinforcing the intestinal barrier and modulating the immunological tone. This approach addresses a critical challenge in current microbiome-based therapies: high inter-individual variability in probiotic colonization efficiency, which is often exacerbated by host-specific colonization resistance [9,49]. By enhancing the mucosal integrity and functional capacity, multiherbal formulations may shift the gut environment toward a more permissive ecological state [51], thereby facilitating the engraftment and functional persistence of both commensal and supplemental microbiota. Accordingly, this integrated strategy offers a testable hypothesis, whereby host-directed modulation by herbal medicine could enhance the clinical consistency and durability of probiotic effects in AAD [52]. Given the long-standing clinical use and well-documented safety profiles of the botanical formulations examined, these integrated approaches represent promising alternatives to single-modality interventions. Furthermore, this concept aligns with the emerging paradigm of “Precision Microbiome” therapy, in which targeted modulation of the host–microbe interface is increasingly recognized as a means of achieving individualized and context-dependent therapeutic outcomes [53]. By optimizing the intestinal environment using botanical primers, future strategies may move toward a more effective and personalized management of antibiotic-associated diarrhea.

### 4.6. Study Limitations

This study has several limitations that should be acknowledged. First, most of the included studies compared multiherb interventions with untreated AAD model controls, with a relative paucity of active comparators such as standard probiotics or fecal microbiota transplantation. Second, substantial heterogeneity in antibiotic induction regimens, treatment durations, and microbiome analytical platforms (e.g., 16S rRNA sequencing versus shotgun metagenomics) precluded formal quantitative meta-analysis, restricting the present synthesis to a qualitative and descriptive framework.

Third, although cluster-specific outcome patterns were identified, dose–response relationships and optimal therapeutic windows remain poorly defined across formulations. Moreover, although a “soil-priming” framework is proposed, most studies did not directly quantify absolute microbial abundance or colonization efficiency, limiting direct validation of enhanced microbial engraftment. In addition, incomplete reporting of randomization and blinding contributed to an overall moderate risk of bias in several domains (Figure 2). The relatively small number of included studies may limit the robustness of the synthesis. Overlap in core herbal components across formulation clusters may complicate cluster-specific interpretations, as these clusters reflect predominant compositional patterns rather than strictly distinct categories. Furthermore, the inclusion was restricted to studies reporting microbiome-related outcomes to ensure a focused analysis of microbiome–host interactions. While this aligns with the primary objective of this review, it may inherently emphasize these specific biological pathways over others. This may also contribute to the observed directional consistency across studies.

Analytical approaches such as network meta-analysis or component-based regression could, in theory, help disentangle the contributions of individual herbs. However, such analyses were not feasible in this review because of the extensive overlap in the core botanical components and the scarcity of studies directly comparing single-herb interventions with their parent formulations. The inherent complexity of herb–herb interactions in traditional decoctions further suggests that therapeutic effects are often synergistic rather than additive, complicating statistical deconstruction without the risk of oversimplification.

Despite these limitations, the current evidence, which is largely derived from animal models, provides an essential mechanistic foundation for future translational research. The cluster-specific therapeutic patterns identified herein suggest that herbal interventions may be tailored to distinct dysbiotic profiles, such as pathogen-dominant and barrier-compromised states. In this context, the proposed soil-priming framework supports the design of future three-arm clinical trials comparing herbal monotherapy, probiotic monotherapy, and their combined administration to rigorously evaluate their synergistic effects in human AAD.

Overall, this review offers a structured synthesis of complex preclinical evidence and highlights recurrent botanical microbiome patterns that may guide future mechanistic and clinical investigations. Given the long-standing clinical use and favorable safety profiles of these botanical formulations, further evaluation of multiherbal strategies as host-directed interventions for AAD is warranted.

## 5. Conclusions

This review highlights that multiherbal formulations in AAD models exert convergent host-directed effects that extend beyond simple microbial replacements. These effects include the reinforcement of the integrity of the intestinal barrier, attenuation of mucosal inflammation, and facilitation of endogenous microbiome recovery. Cluster-based synthesis has revealed distinct therapeutic strategies that converge on the stabilization of the host–microbe interface. Although the currently available evidence is limited to heterogeneous preclinical studies, these findings provide a biologically plausible rationale for integrated herbal–probiotic approaches, conceptualized as a host-directed “soil-priming” strategy that optimizes the intestinal niche for microbial resilience. Collectively, this structured synthesis informs the rational design of future translational studies and clinical trials aimed at improving the durability and consistency of therapeutic outcomes for antibiotic-associated diarrhea.

## Figures and Tables

**Table 1:** Characteristics of animal studies investigating multiherb formulations for antibiotic-associated diarrhea.

Cluster	Study	Formula	Herbal Composition (As Reported)	Animal	AAD Induction	Comparator Arms	Tx Period	Total	Platform
QWBZP-based	[13]	QWBZP	*Atractylodes macrocephala*, *Poria cocos*, *Codonopsis pilosula*, *Glycyrrhiza uralensis*, *Dioscorea opposita*, *Dolichos lablab*, *Nelumbo nucifera*	KM mouse	Gentamicin + cefradine, 5 d	NC/AAD	3 d	8 d	Lactase gene (PCR, cloning)
QWBZP-based	[14]	QWBZP	*Atractylodes macrocephala, Poria cocos, Glycyrrhiza uralensis, Dolichos lablab, Platycodon grandiflorus, Amomum villosum, Aucklandia lappa*	KM mouse	Antibiotic cocktail, 7 d	NC/AAD	7 d	14 d	16S rRNA sequencing
QWBZP-based	[15]	QWBZP glycosides	Derived from *Atractylodes macrocephala, Poria cocos, Glycyrrhiza uralensis, Dolichos lablab, Platycodon grandiflorus, Amomum villosum, Aucklandia lappa*	Mouse	Antibiotics, 7 d	NC/AAD	7 d	14 d	16S rRNA sequencing
QWBZP-based	[16]	QWBZP + sucrose	*Atractylodes macrocephala, Poria cocos, Glycyrrhiza uralensis, Dolichos lablab, Platycodon grandiflorus, Amomum villosum, Aucklandia lappa*	Mouse	Antibiotics, 7 d	NC/AAD	7 d	14 d	16S rRNA sequencing
LZT-based	[17]	LZT	*Panax ginseng*, *Atractylodes macrocephala*, *Zingiber officinale*, *Glycyrrhiza uralensis*	SD rat	Antibiotics, 7 d	NC/AAD	7 d	14 d	Butyrate-related gene (PCR)
LZT-based	[18]	LZT	*Panax ginseng, Atractylodes macrocephala, Zingiber officinale, Glycyrrhiza uralensis*	Rat (♂/♀)	Antibiotics, 7 d	NC/AAD	7 d	14 d	16S rRNA sequencing
LZT-based	[19]	LZT	*Panax ginseng, Atractylodes macrocephala, Zingiber officinale, Glycyrrhiza uralensis*	Mouse	Antibiotics, 7 d	NC/AAD/Magnolia	7 d	14 d	T-RFLP
LZT-based	[20]	LZT (polysaccharide)	Derived from *Panax ginseng, Atractylodes macrocephala, Zingiber officinale, Glycyrrhiza uralensis*	Mouse	AB 7 d + CDI 7 d	NC/AAD	7 d	21 d	16S rRNA sequencing
GQT-based	[21]	GQT	*Pueraria lobata*, *Scutellaria baicalensis*, *Coptis chinensis*, *Glycyrrhiza uralensis*	Rat	Antibiotics, 7 d	NC/AAD	7 d	14 d	16S rRNA sequencing
GQT-based	[22]	GQT	*Pueraria lobata, Scutellaria baicalensis, Coptis chinensis, Glycyrrhiza uralensis*	Rat	Antibiotics, 7 d	NC/AAD	7 d	14 d	Shotgun metagenomics
Coptis-containing but non-identical formulations	[23]	Xianglian Pill	*Coptis chinensis, Aucklandia lappa*	Mouse	Antibiotics, 7 d	NC/AAD	7 d	14 d	16S rRNA sequencing
Coptis-containing	[24]	Shengjiang Xiexin Decoction	*Zingiber officinale, Pinellia ternata, Scutellaria baicalensis, Coptis chinensis, Glycyrrhiza uralensis*	Mouse	*Clostridioides difficile* model	NC/AAD	7 d	14 d	Metabolomics + microbiome
Coptis-containing	[25]	Shengjiang Xiexin Decoction	*Zingiber officinale, Pinellia ternata, Scutellaria baicalensis, Coptis chinensis, Glycyrrhiza uralensis*	Mouse	Antibiotics, 7 d	NC/AAD	7 d	14 d	16S rRNA sequencing
Other	[26]	Hetiao Jianpi Decoction	*Codonopsis pilosula, Atractylodes macrocephala, Poria cocos, Citrus reticulata, Glycyrrhiza uralensis*	SD rat	Antibiotics, 7 d	NC/AAD	7 d	14 d	16S rRNA sequencing
Other multiherb formulations	[27]	TCM ± fermentation	*Atractylodes macrocephala, Poria cocos, Glycyrrhiza uralensis, Citrus reticulata* (fermented vs. non-fermented)	Mouse	Ceftriaxone, 7 d	NC/AAD/FTCM	7 d	14 d	16S rRNA sequencing
Other	[28]	Butuyajie formula	*Atractylodes macrocephala, Poria cocos, Glycyrrhiza uralensis, Citrus reticulata*	Rat	Lincomycin, 7 d	NC/AAD	7 d	14 d	16S rRNA sequencing
Other	[29]	Zisu Tang (gel)	*Perilla frutescens, Glycyrrhiza uralensis*	Mouse	Lincomycin, 5 d	NC/AAD	7 d	12 d	16S rRNA sequencing

Formulation clusters are defined based on recurrent co-occurrence of core herbal components, reflecting shared prescription logic rather than isolated botanical effects. The included formulations are categorized into QWBZP-based, LZT-based, GQT-based, and other supportive multiherb clusters. Abbreviations: AAD, antibiotic-associated diarrhea; QWBZP, Qiwei Baizhu San; LZT, Lizhong Tang; GQT, Gegen Qinlian Tang; and NR, not reported.

**Table 2:** Frequency of key botanical components and their strategic roles in AAD formulations.

Category	Core Botanical Components	Frequency, n (%)	Strategic Role and Rationale
Spleen-Tonifying and Functional Recovery	*Atractylodes macrocephala*	12 (70.6%)	Soil Conditioning: Enhancing “Spleen” transformative functions; restoration of digestive enzymes (e.g., Lactase) and nutrient absorption.
	*Panax ginseng/Codonopsis pilosula*	9 (52.9%)	
	*Poria cocos*	10 (58.8%)	
Warming and Metabolic Regulation	Zingiberis Rhizoma *(Dried/Fresh Ginger)*	7 (41.2%)	Thermal Regulation: “Warming the Middle” to counteract antibiotic-induced cold-type functional suppression and restore α-diversity.
Heat-Clearing and Pathogen Control	*Coptis chinensis*	5 (29.4%)	Detoxification: “Clearing Heat” to eliminate inflammatory “toxins” and inhibit opportunistic pathogens like *Proteobacteria*.
	*Scutellaria baicalensis*	4 (23.5%)	
	*Pueraria lobata*	3 (17.6%)	
Qi-Regulating and Barrier Protection	*Citri Unshius Pericarpium*	4 (23.5%)	Harmonizing and Protecting: Promoting Qi flow to resolve stagnation, reinforce the mucosal barrier (MUC2), and prevent bacterial translocation.
	*Aucklandia lappa/Agastache rugosa*	3 (17.6%)	
Harmonizing and Trophicity	*Glycyrrhiza uralensis*	11 (64.7%)	Coordinating Formulae: “Harmonizing all drugs” and tonifying blood to stabilize the host-microbiome axis and mitigate acute mucosal damage.

Abbreviations: AAD, antibiotic-associated diarrhea; SCFA, short-chain fatty acids. Values represent the number and percentage of studies in which each botanical component is identified.

**Table 3:** Microbiome- and host-related outcomes across individual studies.

Cluster	Study	α-Diversity	β-Diversity	Key Taxa Changes (Genus Level When Available)	SCFAs	Barrier-Related Outcomes	Immune/Inflammatory Markers	Enzyme Activity
QWBZP-based	[13]	↓	NR	Lactase activity ↑; lactase gene abundance ↑	NR	NR	NR	Lactase ↑
QWBZP-based	[14]	↑	Shift vs. AAD	*Lactobacillus* ↑	NR	NR	NR	NR
QWBZP-based	[15]	↑	Shift vs. AAD	BA-associated taxa ↑	NR	NR	NR	NR
QWBZP-based	[16]	↑	Dose-dependent	SCFA-associated taxa ↑	Directly measured (GC-MS) ↑	MUC2 ↑	IL-17 ↓	NR
LZT-based	[17]	NR	NR	Butyrate-producing bacteria ↑	NR	NR	NR	NR
LZT-based	[18]	↑	Shift vs. AAD	Proteobacteria ↓	NR	Barrier repair	Cytokines ↓	NR
LZT-based	[19]	↔/↑	OTU recovery	Firmicutes ↑	NR	NR	NR	NR
LZT-based	[20]	↑	Shift vs. CDI	*C. difficile* ↓; *Bacteroidetes* ↑	NR	NR	NR	NR
GQT-based	[21]	↑	Shift vs. AAD	Beneficial genera ↑	NR	NR	NR	NR
GQT-based	[22]	NR	Functional shift	Functional pathways altered; ARG ↓	NR	NR	NR	NR
Coptis-containing	[23]	↑	Clear separation	*Lactobacillus* ↑	Directly measured (GC-MS) ↑	ZO-1 (protein) ↑	TNF-α ↓	NR
Coptis-containing	[24]	NR	Distinct clustering	Metabolite-associated taxa	NR	NR	NR	NR
Coptis-containing	[25]	↑	Shift vs. AAD	Metabolic taxa ↑	NR	NR	Inflammation ↓	NR
Other	[26]	↑	Shift vs. AAD	*Bacteroides* ↑	NR	NR	NR	NR
Other	[27]	↑	FTCM > TCM	*Lactobacillus* ↑	NR	ZO-1, Occludin (mRNA) ↑	NR	NR
Other	[28]	↑	Shift vs. AAD	*Firmicutes* ↑	NR	Bacterial translocation ↓; DAO ↓; LPS ↓	MPO ↓	NR
Other	[29]	↑	Gel > decoction	Proteobacteria ↓; Lactase ↑	NR	NR	NR	Sucrase ↑

NR indicates that the outcome is not assessed or not reported in the original study and does not imply the absence of an effect. Formulation clusters are defined based on recurrent co-occurrence of core herbal components, reflecting shared prescription logic rather than isolated botanical effects. The included formulations are categorized into QWBZP-based, LZT-based, GQT-based, and other supportive multiherb clusters. Abbreviations: AAD, antibiotic-associated diarrhea; CDI, clostridioide difficile infection; SCFA, short-chain fatty acid; OTU, operational taxonomic unit; TCM, traditional Chinese medicine; TFCM, fermented traditional Chinese medicine; NR, not reported. ↑, increase; and ↓, decrease.

**Table 4:** Integrated outcome patterns across formulation clusters in AAD animal models.

Outcome Domain	QWBZP-Based (n = 4)	Lizhong-Based (n = 4)	GQT-Based (n = 2)	Coptis-Containing (n = 3)	Other Multiherb (n = 4)
α-diversity	↑ in 3/4; ↓ in 1/4 [13]	↑ (recovery) in 3/4; NR in 1/4	↑ (recovery) in 1/2; NR in 1/2	↑ (recovery) in 2/3; NR in 1/3	↑ (recovery) in 4/4
β-diversity	Shift vs. AAD (2/4); NR (2/4)	Restored in 3/4; NR in 1/4	Restored in 2/2	Distinct clustering (3/3)	Shift vs. AAD (4/4)
Key taxa (genus-level when available)	*Lactobacillus* ↑ (3/4); Lactase-pos. bacteria ↑ [13]	*Firmicutes* ↑ (2/4); *Proteobacteria* ↓ (2/4)	Beneficial genera ↑ [21]; Functional shift (1/2)	*Lactobacillus* ↑ (2/3); Metabolic taxa ↑ (2/3)	*Firmicutes* ↑ (3/4); *Proteobacteria* ↓ (3/4)
SCFA (direct measurement)	NR (4/4)	NR (4/4)	NR (2/2)	Measured (GC-MS) in 1/3 [23]	Measured (GC-MS) in 1/4 [16]
Barrier-related outcomes	NR (4/4)	Barrier repair (1/4 [18]); NR in 3/4	NR (2/2)	ZO-1/Occludin ↑ (2/3); NR in 1/3	Bacterial translocation ↓ [28]
Immune/inflammatory markers	NR (4/4)	Cytokines ↓ (1/4 [18]); NR in 3/4	NR (2/2)	TNF-α ↓; IL-17 ↓ (2/3); NR in 1/3	MPO ↓ (1/4 [28]); NR in 3/4
Digestive enzyme activity	Lactase ↑ (1/4 [13]); NR in 3/4	NR (4/4)	NR (2/2)	NR (3/3)	Sucrase/Lactase ↑ (1/4 [29]); NR in 3/4
Analytical platform	Functional restoration of digestion	Microbiota rebalancing and metabolism	Anti-inflammatory and metabolic regulation	Mucosal and immune modulation	Broad dysbiosis correction

Abbreviations: AAD, antibiotic-associated diarrhea; QWBZP, Qiwei Baizhu San; LZT, Lizhong Tang; GQT, Gegen Qinlian Tang; SCFA, short-chain fatty acid; GC–MS, gas chromatography–mass spectrometry; NR, not assessed or not reported; MPO, myeloperoxidase; TNF-α, tumor necrosis factor-alpha; IL-17, interleukin-17. ↑, increase; and ↓, decrease.

## Data Availability

No new data were created or analyzed in this study. Data sharing is not applicable to this article.
